# On Polypus of the Rectum

**Published:** 1847-03

**Authors:** Robert Burns

**Affiliations:** Frankford, Pennsylvania


*On Polypus of the Rectum.* By Robert Burns, M. D., Frankford, Pennsylvania.

To the Editor of the Medical Examiner.

Sir,—On perusing the January number of the Examiner, my attention was drawn particularly to an article among your medical records, upon *Polypus of the Rectum,* which recalled to my remembrance a case of this kind, which came under my care in the early part of the past summer; and should the following notice of the same be thought worth an insertion in your journal, it is at your disposal.

E. K. Y——s was from her birth what her mother termed a very troublesome child, being frequently indisposed. When about two and a half or three years old, her mother observed that she suffered much distress previous to the act of defecation, and before it could be accomplished, there was a protrusion of a dark red fleshy substance *ex ano,* which bled less or more on every occasion of the kind. This tumour was shortly associated with a second of smaller size, which, after remaining some months, separated from the larger one; this latter continued to enlarge, until, according to the mother's description, it had acquired the size of a small hen's egg. She states it was exceedingly troublesome, requiring to be replaced within the sphincter ani after every evacuation. This prolapse of the polypus always took place before the evacuation of the intestine, and never went up without manual assistance. This state of things continued up to the age of five years, when I was consulted upon the case. At this time the child was considerably reduced by sanguineous discharge. She had a distressed, anxious appearance, with great timidity resulting from the knowledge of her infirmity, and apparent consciousness that others knew it also. This rendered her an object of deep consideration and sympathy, apart from the usual and necessary interest which must be felt in every case of suffering humanity by the medical practitioner. The physical development of this child was naturally good, her eyes and skin dark, hair brown, and countenance open and intelligent.

On examination of the anus, I found a polypus protruding as large as a small seckel pear, being considerably diminished at the time from frequent bleedings. On dilating the anus as much as possible, by traction in opposite directions, I found the pedicle to be somewhat elongated from the mucous membrane of the rectum above the sphincter; to this I immediately applied a ligature of silk thread, allowing the ends to remain long, which were secured externally by a strip of adhesive plaster; about twenty-four hours after its application the polypus separated, and two days after, the ligature came away. She suffered some pain while the ligature remained, which did not demand special attention. Since its removal, about eight months have passed away, and there has been no return of the disease; no hemorrhage, the function of the part is normal, and the child's health and appearance very much improved.

